# Supplementary material for: RNA secondary structure prediction using deep learning with thermodynamic integration
Source: Nat Commun. 2021 Feb 11;12:941. doi: 10.1038/s41467-021-21194-4 (PMC7878809; doi:10.1038/s41467-021-21194-4)
Supplement: Supplementary file 1 — Supplementary Information [file 41467_2021_21194_MOESM1_ESM.pdf]

Supplementary information for “RNA secondary structure prediction using deep learning with thermodynamic integration”

Kengo Sato[1,*], Manato Akiyama[1], and Yasubumi Sakakibara[1]

[1] Department of Biosciences and Informatics, Keio University, 3–14–1 Hiyoshi, Kohoku-ku, Yokohama 223–8522, Japan.
[*]To whom correspondence should be addressed.

Suppelemtary Methods

Zuker-style dynamic programming algorithm

We assume that a given RNA secondary structure can be decomposed into nearest-neighbor loop substructures according to the Turner's nearest-neighbor model and that the free energy of the given RNA secondary structure can be calculated by summing the free energy of every nearest-neighbor loop.

Since it is not practical to enumerate all the secondary structures that can be folded into, we use a dynamic programming technique to calculate the secondary structure with the minimum free energy, which is also known as the Zuker algorithm [6].

We consider that $x_{i:j} = x_i, \ldots, x_j$, a subsequence of $x = x_1, \ldots, x_n$, is decomposed into shorter subsequences based on the loop types. If $x_{i:j}$ is an external loop F that is not closed by any base pairs, then it is recursively decomposed until finding a closed loop. A closed loop C closed by a base pair (i, j) is decomposed in one of the following three ways, depending on the number of closing base pairs. If the closed loop C is closed only by the base pair (i, j), it is a hairpin loop. If C is closed by two base pairs (i, j) and (k, l) ($i < k$ and $j > l$), it is decomposed into either a stacking (for $k = i + 1$ and $l = j - 1$), a bulge loop (for $k = i + 1$ or $l = j - 1$) or an internal loop (for $k > i+1$ and $l < j-1$). A loop closed by a base pair (i, j) and two or more bases pairs inside (i, j) is called a multibranch loop. The multibranch loop is decomposed into the rightmost loop M^1 and the other loops M, where M^1 contains exactly one closed loop C, and M contains one or more loops C, disambiguating the decomposition of RNA secondary structure.

Based on the decomposition described above, the recursive equation for the dynamic programming algorithm to calculate the minimum free energy is as follows:

$$
\begin{aligned}
F_{ij} &= \min\left\{F_{i+1,j}, \min_{i<k\leq j} C_{ik} + F_{k+1,j}\right\} \\
C_{ij} &= \min\left\{\mathcal{H}(i,j), \min_{i<k<l<j} C_{kl} + \mathcal{I}(i,j;k,l), \min_{i<u<j} M_{i+1,u} + M^1_{u+1,j-1} + a\right\} \\
M_{ij} &= \min\left\{\min_{i<u<j}(u-i+1)c + C_{u+1,j} + b, \min_{i<u<j} M_{iu} + C_{u+1,j} + b, M_{i,j-1} + c\right\} \\
M^1_{ij} &= \min\left\{M^1_{i,j-1} + c, C_{ij} + b\right\} \\
F_{ii} &= 0, C_{ii} = M_{ii} = M^1_{ii} = \infty,
\end{aligned}
\tag{1}
$$

where F_{ij} is the minimum free energy of the secondary structure of the subsequence $x_{i:j}$, and C_{ij} represents the MFE over closed structure. M_{ij} is the MFE of part of a multibranch loop that contains one or more loops, and M^1_{ij} is the MFE of part of a multibranch loop with the rightmost loop.

Here, $\mathcal{H}(i, j)$ is the free energy of a hairpin loop $x_{i:j}$ closed by the base pair (i, j). $\mathcal{I}(i, j; k, l)$ is the free energy of a stacking (for $k = i+1$ and $l = j-1$), a bulge loop (for $k = i+1$ or $l = j-1$), or an internal loop (otherwise) closed by the base pairs (i, j) and (k, l). The free energy of multibranch loop is approximated using parameters a, b and c as follows:

$$a + b \times (\text{\# of base pairs}) + c \times (\text{\# of unpaired bases})$$

For the thermodynamic-based methods, these loop energies are calculated by using the Turner's nearest-neighbor free energy parameters pre-determined by experimental methods such as the optical melting analysis. As an alternative to the thermodynamic parameters, our algorithm calculates the free energy of the nearest-neighbor loops using four types of folding scores computed by the deep neural network: helix stacking scores, helix opening scores, helix closing scores, and unpaired region scores.

The algorithm starts with the smallest subsequences, i.e., empty strings, then fills F_{ij}, C_{ij}, M_{ij}, and M^1_{ij} by using Eq. (1), and ends up with $F_{1,n}$ that will be filled by MFE. The algorithm requires $O(n^3)$ time for calculating C_{ij} of the free energy of the internal loops closed by the base pairs (i, j) and (k, l). However, it can be reduced to $O(n^3)$ time by limiting the number of unpaired bases that form interal loops as L (generally $L = 30$). The MFE structure can be recovered by tracing back the recursive equations applied to construct MFE from $F_{1,n}$.

Supplementary Figures

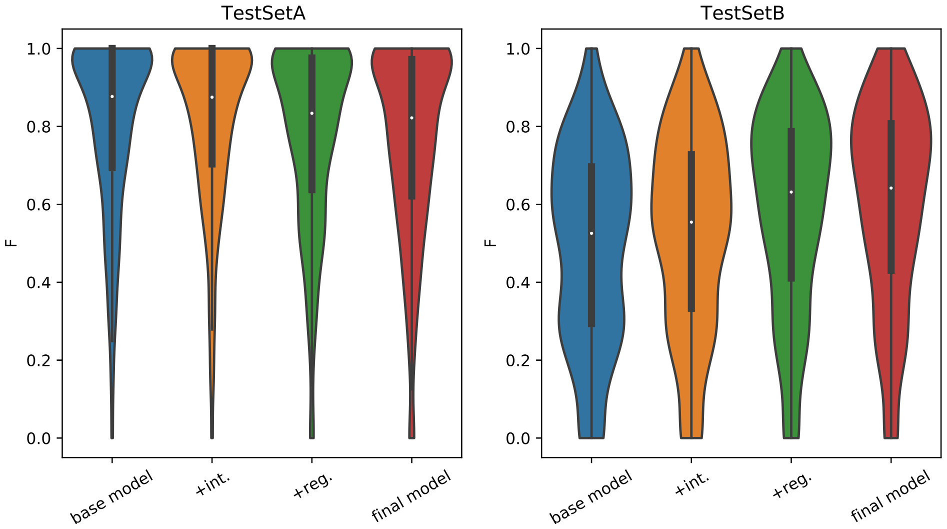

Supplementary Figure 1: F-values of the base model and the use of the thermodynamic-related techniques. +int.: the use of the thermodynamic-integrated k scores, +reg.: the use of the thermodynamic regularization.

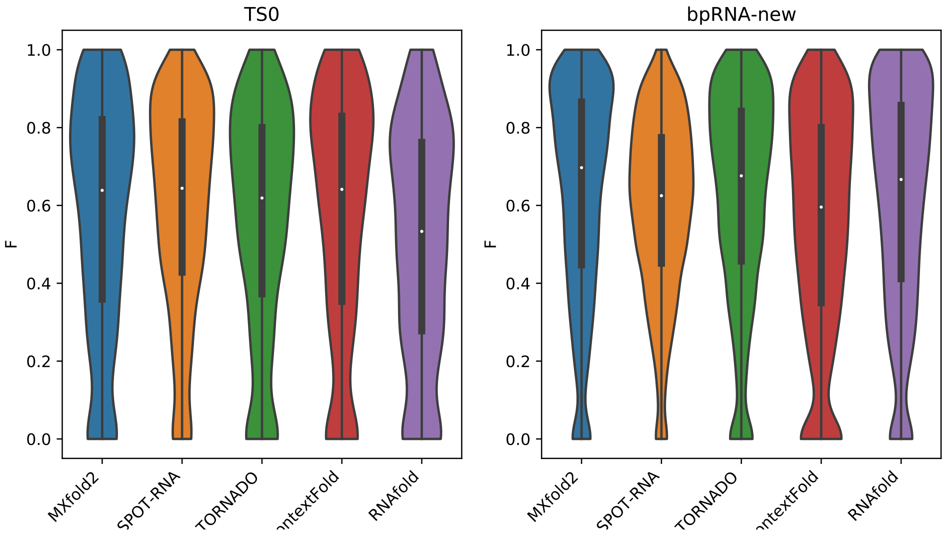

Supplementary Figure 2: F-values of MXfold2, SPOT-RNA, TORNADO, ContextFold, and RNAfold on the TS0 dataset for sequence-wise cross-validation (CV) and the bpRNA-new dataset for family-wise CV. All trainable methods were trained with the TR0 dataset.

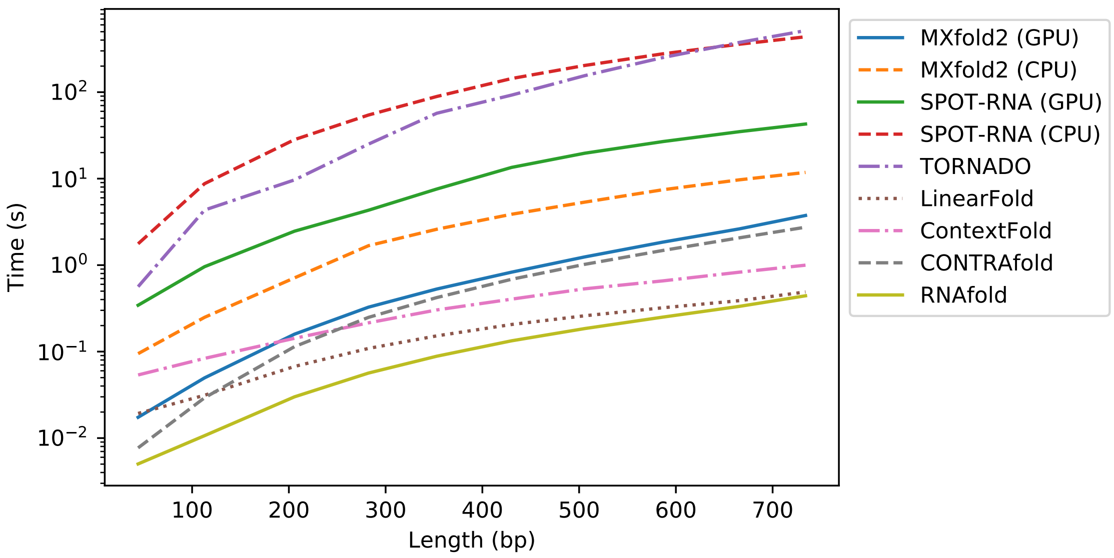

Supplementary Figure 3: The running time for the lengths of input sequences in TestSetA measured on Linux OS v4.15.0 with Intel XeonE5-2698v4 (2.20 GHz) and NVIDIA Tesla V100.

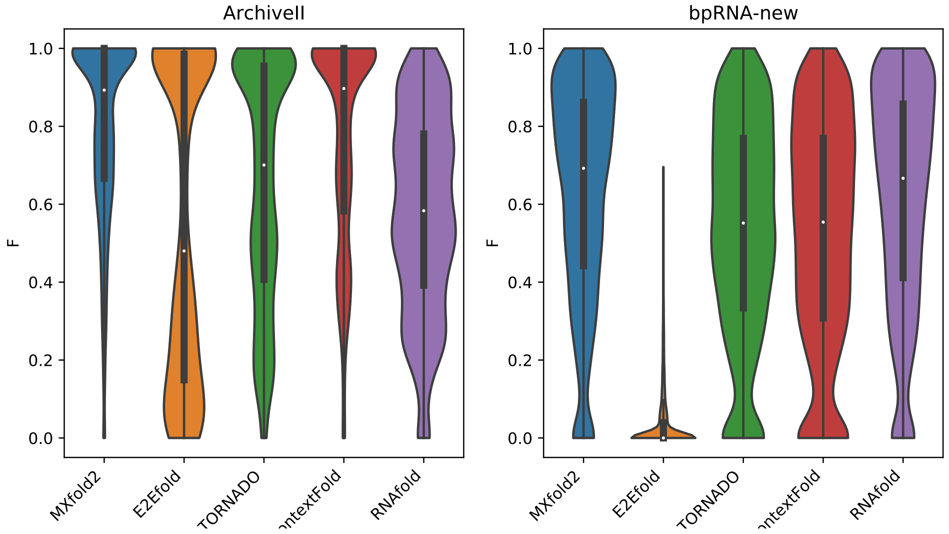

Supplementary Figure 4: F-values of MXfold2, E2Efold, TORNADO, ContextFold, and RNAfold using the ArchiveII dataset for sequence-wise cross-validation (CV) and the bpRNA-new dataset for family-wise CV. All trainable methods were trained using the RNAStrAlign dataset.

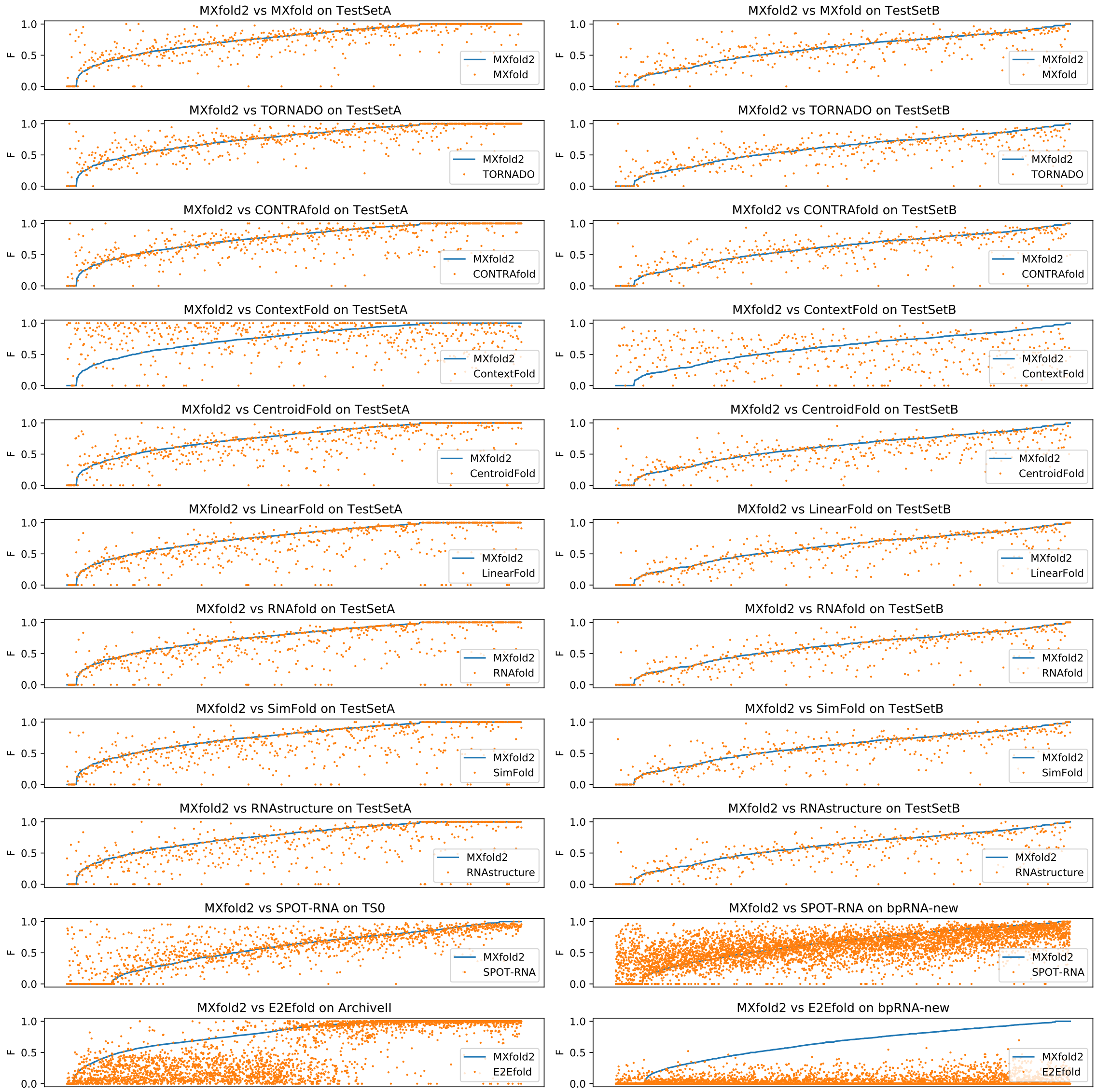

Supplementary Figure 5: Comparison of F values between MXfold2 and each competitive method for each sequence. All sequences are ranked by the F values of MXfold2, and MXfold2 is compared with each competitive method for each sequence. The plots in the left and right columns are comparisons in the sequence-wise and family-wise cross-validation, respectively.

Supplementary Tables

Supplementary Table 1: The summary of datasets used in our experiments.

		# sequences	length	source
Rivas Dataset	TrainSetA	3,166	10–734	Rivas *et al.* [3]
	TestSetA	592	10–768	
	TestSetB	430	27–244	
bpRNA-1m	TR0	10,814	33–498	Danaee *et al.* [2]
	TS0	1,305	22–499	
bpRNA-new		5,401	33–489	the present study
RNAStrAlign	train	20,923	30–600	Tan *et al.* [5]
ArchiveII		3,966	28–1,800	Sloma *et al.* [4]
T-Full		1,291	9–55	Andronescu *et al.* [1]

Supplementary Table 2: Comparison of the accuracy of the secondary structure prediction with the existing methods using TestSetA.

	PPV	Sen	F	p
CONTRAfold	0.708	0.745	0.719***	2.0×10^{-8}
CentroidFold	0.701	0.678	0.678***	4.1×10^{-26}
ContextFold	0.777	0.750	**0.759**	0.49
LinearFold	0.658	0.667	0.642***	9.3×10^{-41}
MXfold	0.768	0.731	0.739***	6.5×10^{-4}
MXfold2	0.754	0.778	**0.761**	—
RNAfold	0.658	0.668	0.642***	9.8×10^{-41}
RNAstructure	0.657	0.650	0.631***	5.7×10^{-44}
SimFold	0.654	0.643	0.629***	5.8×10^{-44}
TORNADO	0.749	0.754	0.746***	9.2×10^{-4}

All trainable methods were trained using TrainSetA. F-values that are significantly worse than the best are marked with * ($p < 0.05$), ** ($p < 0.01$) and *** ($p < 0.001$) as calculated with one-sided Wilcoxon signed-rank test. Others are in bold.

Supplementary Table 3: Comparison of the accuracy of the secondary structure prediction with the existing methods using TestSetB.

	PPV	Sen	F	p
CONTRAfold	0.530	0.640	0.573^{***}	2.9×10^{-4}
CentroidFold	0.497	0.556	0.518^{***}	7.3×10^{-22}
ContextFold	0.485	0.534	0.502^{***}	2.8×10^{-8}
LinearFold	0.501	0.609	0.544^{***}	1.5×10^{-12}
MXfold	0.561	0.620	0.582^{*}	0.027
MXfold2	0.571	0.650	**0.601**	—
RNAfold	0.498	0.606	0.540^{***}	4.9×10^{-16}
RNAstructure	0.480	0.588	0.522^{***}	5.6×10^{-19}
SimFold	0.512	0.611	0.551^{***}	1.6×10^{-11}
TORNADO	0.528	0.594	0.552^{***}	5.1×10^{-7}

See the footnote of Supplementary Table 2.

Supplementary Table 4: Comparison of the accuracy of the secondary structure prediction with the existing methods using the combined dataset with TestSetA and TestSetB.

	PPV	Sen	F	p
CONTRAfold	0.633	0.701	0.658^{***}	7.4×10^{-11}
CentroidFold	0.615	0.627	0.611^{***}	5.8×10^{-46}
ContextFold	0.654	0.659	0.651^{***}	9.7×10^{-5}
LinearFold	0.592	0.643	0.600^{***}	1.7×10^{-49}
MXfold	0.681	0.684	0.673^{***}	1.5×10^{-4}
MXfold2	0.677	0.724	**0.693**	—
RNAfold	0.590	0.642	0.599^{***}	8.8×10^{-54}
RNAstructure	0.582	0.624	0.585^{***}	1.7×10^{-60}
SimFold	0.594	0.629	0.596^{***}	6.3×10^{-52}
TORNADO	0.656	0.687	0.664^{***}	9.5×10^{-9}

See the footnote of Supplementary Table 2.

Supplementary Table 5: Comparison of the accuracy of the secondary structure prediction among MXfold2, E2Efold, TORNADO, ContextFold, and RNAfold.

	Sequence-wise CV[1]			Family-wise CV[2]		
	PPV	SEN	F	PPV	SEN	F
MXfold2[3]	0.790	0.815	0.800	0.575	0.712	0.628
E2Efold[3]	0.605	0.519	0.548	0.0474	0.0307	0.0361
TORNADO[3]	0.643	0.661	0.649	0.500	0.588	0.532
ContextFold[3]	0.770	0.771	0.768	0.559	0.506	0.522
RNAfold	0.551	0.613	0.577	0.552	0.720	0.617

[1] Sequence-wise cross-validation (CV) with the ArchiveII dataset.
[2] Family-wise CV with the bpRNA-new dataset.
[3] All trainable methods were trained using the RNAStrAlign dataset.

Supplementary References

[1] M. Andronescu, A. Condon, H. H. Hoos, D. H. Mathews, and K. P. Murphy. Computational approaches for RNA energy parameter estimation. *RNA*, 16(12):2304–2318, Dec. 2010.

[2] P. Danaee, M. Rouches, M. Wiley, D. Deng, L. Huang, and D. Hendrix. bpRNA: large-scale automated annotation and analysis of RNA secondary structure. *Nucleic Acids Res.*, 46(11):5381–5394, June 2018.

[3] E. Rivas, R. Lang, and S. R. Eddy. A range of complex probabilistic models for RNA secondary structure prediction that includes the nearest-neighbor model and more. *RNA*, 18(2):193–212, Feb. 2012.

[4] M. F. Sloma and D. H. Mathews. Exact calculation of loop formation probability identifies folding motifs in RNA secondary structures. *RNA*, 22(12):1808–1818, Dec. 2016.

[5] Z. Tan, Y. Fu, G. Sharma, and D. H. Mathews. TurboFold II: RNA structural alignment and secondary structure prediction informed by multiple homologs. *Nucleic Acids Res.*, 45(20):11570–11581, 2017.

[6] M. Zuker and P. Stiegler. Optimal computer folding of large RNA sequences using thermodynamics and auxiliary information. *Nucleic Acids Res.*, 9(1):133–148, Jan. 1981.
