# Supplementary material for: RNA secondary structure prediction using deep learning with thermodynamic integration
Source: Nat Commun. 2021 Feb 11;12:941. doi: 10.1038/s41467-021-21194-4 (PMC7878809; doi:10.1038/s41467-021-21194-4)
Supplement: Supplementary file 3 — Reporting Summary [file 41467_2021_21194_MOESM3_ESM.pdf]

nature research

Corresponding author(s): Kengo Sato
Last updated by author(s): Jan 10, 2021

Reporting Summary

Nature Research wishes to improve the reproducibility of the work that we publish. This form provides structure for consistency and transparency in reporting. For further information on Nature Research policies, see our Editorial Policies and the Editorial Policy Checklist.

Statistics

For all statistical analyses, confirm that the following items are present in the figure legend, table legend, main text, or Methods section.

n/a	Confirmed	
☐	☒	The exact sample size (*n*) for each experimental group/condition, given as a discrete number and unit of measurement
☒	☐	A statement on whether measurements were taken from distinct samples or whether the same sample was measured repeatedly
☐	☒	The statistical test(s) used AND whether they are one- or two-sided<br>*Only common tests should be described solely by name; describe more complex techniques in the Methods section.*
☒	☐	A description of all covariates tested
☒	☐	A description of any assumptions or corrections, such as tests of normality and adjustment for multiple comparisons
☒	☐	A full description of the statistical parameters including central tendency (e.g. means) or other basic estimates (e.g. regression coefficient) AND variation (e.g. standard deviation) or associated estimates of uncertainty (e.g. confidence intervals)
☐	☒	For null hypothesis testing, the test statistic (e.g. F, t, r) with confidence intervals, effect sizes, degrees of freedom and P value noted<br>*Give P values as exact values whenever suitable.*
☒	☐	For Bayesian analysis, information on the choice of priors and Markov chain Monte Carlo settings
☒	☐	For hierarchical and complex designs, identification of the appropriate level for tests and full reporting of outcomes
☒	☐	Estimates of effect sizes (e.g. Cohen's *d*, Pearson's *r*), indicating how they were calculated

Our web collection on statistics for biologists contains articles on many of the points above.

Software and code

Policy information about availability of computer code

Data collection	The datasets used in our experiments were downloaded from:<br>http://eddylab.org/publications/RivasEddy12/Supplementary_material.tar.gz (Rivas dataset),<br>https://www.dropbox.com/s/w3kc4iro8ztbf3m/bpRNA_dataset.zip (bpRNA TR0 and TS0),<br>https://drive.google.com/drive/folders/19KPRYJjjMJh1qdMhtmUoYA_ncw3ocAHc (RNAStrAlign and ArchiveII), and<br>https://www.cs.ubc.ca/labs/beta/Projects/RNA-Params/results_data_scripts.tar.gz (T-full dataset).<br>To build our original dataset, named bpRNA-new, we downloaded Rfam database 14.2 from https://rfam.xfam.org/.

Data analysis	Predictions of RNA secondary structures were performed by the following software packages:<br>MXfold version 0.0.2 (our previous work),<br>Tornado version 0.3,<br>LinearFold (committed on Feb 5, 2020),<br>CONTRAfold version 2.02,<br>ContextFold version 1.00,<br>CentroidFold version 0.0.16,<br>RNAfold in Vienna RNA package version 2.4.14,<br>SimFold version 2.1,<br>RNAstructure version 6.2,<br>SPOT-RNA (committed on May 28, 2020),<br>E2Efold (committed on Jun 27 , 2020),<br>and our software MXfold2 available at https://github.com/keio-bioinformatics/mxfold2.

For manuscripts utilizing custom algorithms or software that are central to the research but not yet described in published literature, software must be made available to editors and reviewers. We strongly encourage code deposition in a community repository (e.g. GitHub). See the Nature Research guidelines for submitting code & software for further information.

Data

Policy information about availability of data

All manuscripts must include a data availability statement. This statement should provide the following information, where applicable:

- Accession codes, unique identifiers, or web links for publicly available datasets
- A list of figures that have associated raw data
- A description of any restrictions on data availability

All the data sets used in our paper are available at https://doi.org/10.5281/zenodo.4430150.

Field-specific reporting

Please select the one below that is the best fit for your research. If you are not sure, read the appropriate sections before making your selection.

[x] Life sciences [ ] Behavioural & social sciences [ ] Ecological, evolutionary & environmental sciences

For a reference copy of the document with all sections, see nature.com/documents/nr-reporting-summary-flat.pdf

Life sciences study design

All studies must disclose on these points even when the disclosure is negative.

Sample size	The sample size of the bpRNAnew dataset was determined by the number of sequences that were identified as non-redundant by CD-HIT-EST among the 2588 families of seed alignments that were registered after the Rfam version 12.2.
Data exclusions	All redundant sequences homologous to each other were excluded by using CD-HIT-EST version 4.8.1 with 80% sequence identity cutoff.
Replication	All the training weights/parameters of the models were saved during experiments. These trained parameters are deployed at https://github.com/keio-bioinformatics/mxfold2 for results replication.
Randomization	For sequence-wise cross-validation, all the sequences were randomly split into training, test data sets.
Blinding	The test sets used for performance measures were completely independent of data sets used for model training.<br>For sequence-wise cross-validation, CD-HIT-EST tools was used to separate test sets from training data.<br>For family-wise cross-validation, we employed the test sets with completely different families from the training sets.

Reporting for specific materials, systems and methods

We require information from authors about some types of materials, experimental systems and methods used in many studies. Here, indicate whether each material, system or method listed is relevant to your study. If you are not sure if a list item applies to your research, read the appropriate section before selecting a response.

Materials & experimental systems

n/a	Involved in the study
☒	☐ Antibodies
☒	☐ Eukaryotic cell lines
☒	☐ Palaeontology and archaeology
☒	☐ Animals and other organisms
☒	☐ Human research participants
☒	☐ Clinical data
☒	☐ Dual use research of concern

Methods

n/a	Involved in the study
☒	☐ ChIP-seq
☒	☐ Flow cytometry
☒	☐ MRI-based neuroimaging
